# Incidence of Bleeding‐Related Complications During Primary Implantation and Replacement of Cardiac Implantable Electronic Devices

**DOI:** 10.1161/JAHA.116.004263

**Published:** 2017-01-22

**Authors:** Christine I. Nichols, Joshua G. Vose

**Affiliations:** ^1^ Medtronic Advanced Energy Portsmouth NH

**Keywords:** complications, electrophysiology, hemorrhage, infection, pacemakers, Electrophysiology, Pacemaker, Complications, Quality and Outcomes

## Abstract

**Background:**

Use of cardiac implantable electronic devices (CIEDs) is increasing. The incidence of bleeding‐related complications during CIED procedures and the association with subsequent infection risk have been studied in trial settings but not in nonrandomized “real‐world” populations.

**Methods and Results:**

This retrospective database analysis of US insurance claims from the Truven MarketScan database (2009‐2013) evaluated the incidence of bleeding complications during, or in the 30 days following, a CIED procedure and the association between bleeding and subsequent infection in days 31 to 365 of follow‐up. This study identified 42 606 patients who had a primary or replacement CIED procedure and met all inclusion criteria. Incidence of bleeding ranged from 0.58% to 2.81% by type of pharmaceutical therapy. Incidence of infection during days 31 to 365 of follow‐up was significantly higher among patients with a bleeding complication in the first 30 days versus those without (6.56% vs 1.24%, *P*<0.001), with results upheld in multivariate analysis (HR=2.97, 95% CI 1.94‐4.54, *P*<0.001).

**Conclusions:**

This study provides a lower bound of the real‐world incidence of bleeding complications following a CIED procedure within the coding limitations of an insurance claims database. Results confirm the association between bleeding in the pocket and risk of subsequent infection. Further research is needed to precisely identify the costs associated with bleeding in the pocket.

## Introduction

As the average age of the US population rises, the prevalence of cardiac implantable electronic devices (CIEDs) is also growing. A retrospective analysis of the US Nationwide Inpatient Sample data set found that the incidence of de novo pacemaker and implantable cardioverter defibrillator (ICD) implants increased by 45% and 504%, respectively, between 1993 and 2008.^1^ Concurrently, as the average US citizen's life expectancy has increased by over 8 years between 1960 and 2007,^2^ the incidence of generator replacement procedures has also increased as patients outlive the battery life of their devices. Older models typically have an average battery life of 4 to 5 years, whereas select newer models provide therapy for up to 10 years depending on device type.^3^ Most concerning, however, is that with the rise in the annual number of procedures, the comorbidity burden and case complexity of patients presenting for CIED procedures has increased over the same 2 decades.^4^

Although generator replacement is considered a relatively routine procedure, it remains associated with a meaningful risk of complications, including hematoma, infection, and lead damage.^5^, ^6^ Results from recent retrospective and prospective studies cite a wide range of estimates for the incidence of bleeding complications, ranging from 1.1% to 16%.^7^, ^8^, ^9^, ^10^, ^11^, ^12^, ^13^ Once bleeding occurs, clinically meaningful pocket hematoma has been linked to a significant increase in perioperative pain and decrease in quality of life.^9^ Further, bleeding complications are associated with greater risk of device infection, the need to reopen the pocket for drainage, prolonged hospitalization, and significant increases in medical resource utilization.^9^, ^12^ Specifically, 1‐year follow‐up results from the Bridge or Continue Coumadin for Device Surgery Randomized Controlled Trial (BRUISE CONTROL) showed that the risk of device infection was more than 7‐fold greater among patients with clinically significant pocket hematoma versus those without (*P*<0.001).^14^ Further, results from BRUISE CONTROL advanced the literature beyond previously inconsistent findings on the association between hematoma and infection.^15^, ^16^, ^17^, ^18^, ^19^

Importantly, there is a lack of data regarding the incidence of bleeding‐related complications during and following CIED procedures using real‐world, nonrandomized, data sets. There is 1 published study (to our knowledge) that examined the incidence of bleeding‐related complications and subsequent payer burden using data from the US Nationwide Inpatient Sample data set.^12^ Authors demonstrated that among patients who developed pocket hematoma during a CIED procedure, total hospitalization payments were $14 491 greater compared to patients with no hematoma complication ($48 815 vs $34 324, *P*<0.001).^12^ Older age, more complex CIED type, and history of congestive heart failure or coagulopathies were significantly associated with greater risk of pocket hematoma development. However, this study is limited in its generalizability because the authors used an extremely broad codes list to identify occurrence of bleeding‐related complications, thereby likely capturing other device‐related complications not specific to bleeding events.

Given that the majority of the literature to date has focused on the incidence of bleeding alone, we sought to estimate the association between bleeding and subsequent risk of infection by refining the analysis of the US Nationwide Inpatient Sample data set. Additionally, we attempted to confirm findings of BRUISE CONTROL linking pocket hematoma to infection but now with a nonrandomized real‐world data set. Similar to the main limitation cited by Sridhar et al, there is no definitive diagnosis or procedure code to identify bleeding‐related complications in the pocket.^12^ Thus, with our methodology, this analysis may provide a more representative and precise “lower bound” of the incidence of hematoma observed in real‐world practice than prior analyses. Using a large retrospective claims database of commercially insured and Medicare supplemental patients, we evaluated the incidence of hematoma in the first 30 days following a CIED procedure as well as the association between hematoma and subsequent development of infection.

## Methods

### Study Design, Data Source, Patients, and Study Period

This study was a retrospective database analysis of healthcare claims data from the MarketScan Commercial Research Database (Truven Health Analytics, Ann Arbor, MI) for 2009‐2013. This nationally representative database of patients covered by private health insurance includes information for more than 180 million unique patients. Because the database is fully deidentified, this study did not require institutional review board approval. Patients with Medicare were included in the data set if they had some form of supplemental health insurance coverage.

Based on the hypothesis that the incidence and resource use associated with bleeding would vary by device type, patients who underwent a CIED procedure were grouped into 4 study cohorts: primary pacemaker, replacement pacemaker, primary ICD, and replacement ICD. We did not evaluate cardiac resynchronization therapy pacemakers or cardiac resynchronization therapy defibrillators due to both small sample sizes and imprecise coding to reliably identify these device types in insurance claims data sets. All patients were required to be aged 18 or older and to have continuous health plan enrollment from baseline through follow‐up (with an allowed 1‐month gap in coverage).

Patients were first identified based on the presence of an International Classification of Diseases procedure code or specific combination of Current Procedural Terminology procedure codes for CIED generator implantation, replacement, or removal (Table S1). The date of the outpatient visit for the CIED procedure served as the index date for analysis. Patients undergoing other major cardiac procedures, such as diagnostic procedures on the heart and pericardium, percutaneous coronary intervention, coronary bypass graft surgery, catheter ablation, or heart valve surgery during the index visit were excluded from analysis, similar to the methodology outlined by Reynolds et al, who evaluated the incidence of early complications following ICD implantation using Medicare claims data.^20^

**Figure 1:**
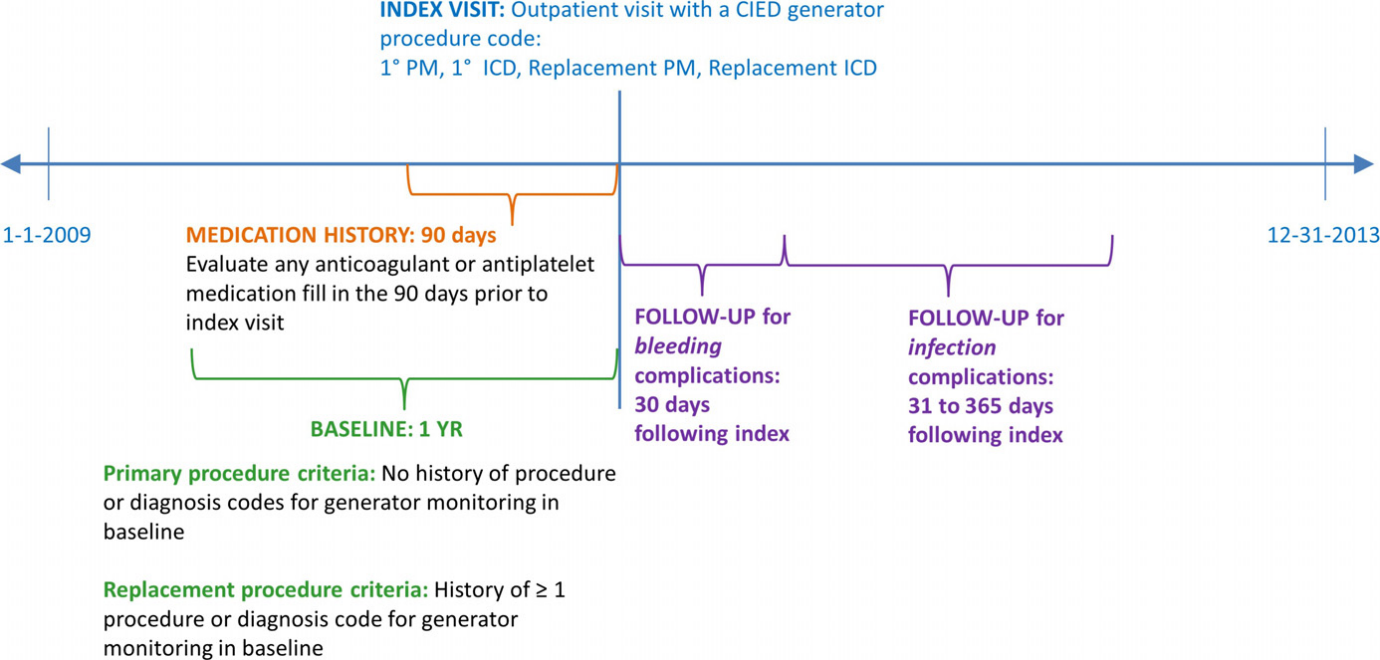
Study period for analysis. CIED indicates cardiac implantable electronic devices; ICD, implantable cardioverter defibrillator; PM, pacemaker.

A 1‐year baseline period was defined as the 12 months prior to the index visit. To ensure that patients were correctly identified as undergoing primary (or replacement) CIED placement procedures, patients were segmented based on the absence (or presence) of diagnosis codes for history of cardiac device or procedure codes for CIED monitoring during the baseline period (Table S2). Due to the relative lack of specificity in US procedure codes prior to 2016, it was not possible to distinguish between 1‐to‐1 device replacements and upgrades and whether complete lead extraction and/or lead addition occurred during the visit. Figure 1 summarizes the study period for this analysis.

### Study Measures

Patient age, sex, and race were summarized as of the index date. Comorbidity status was evaluated by calculating a Charlson Comorbidity Index score for each patient, using diagnoses recorded any time from baseline through follow‐up. The Charlson score is a composite measure of physical health status commonly used in studies of medical claims and chronic disease, principally to predict 10‐year mortality of patients with a range of comorbid conditions.^21^, ^22^

History of pharmaceutical therapy was defined as a prescription fill within 90 days prior to the index visit. This timeframe was selected because it is the longest commonly available supply for anticoagulant or antiplatelet therapies. We did not evaluate medication use over the entire 1‐year baseline in order to limit the inclusion criteria to patients with prescription fills within a closer timeframe to the index procedure of interest. Anticoagulant use was defined as any fill for warfarin or the more recent novel oral anticoagulants including apixaban, rivaroxaban, or dabigatran (excluding edoxaban because US Food and Drug Administration clearance was granted after 2013, the end of our data set). Antiplatelet use was defined as any fill for abciximab, eptifibatide, tirofiban, cilostazol, clopidogrel, dipyridamole, prasugrel, ticagrelor, ticlopidine, or vorapaxar. Aspirin use was only captured if the patient filled a prescription; over‐the‐counter medication use was not available in the data set used for analysis. Medication use was grouped into 4 mutually exclusive groups as follows: warfarin monotherapy, novel oral anticoagulant monotherapies, antiplatelet monotherapy, or concurrent warfarin and antiplatelet therapy (patients were prescribed both therapies).

Bleeding‐related complications during the index visit were defined based on any of the following: diagnosis codes for hemorrhage, hematoma, or seroma complicating a procedure (diagnosis code 998.1x); procedure code for control of hemorrhage following vascular surgery or incision with drainage (procedure codes 39.41, 39.98, 86.04, 10140, 10160); or codes for transfusion (procedure codes 99.00 to 99.04, 36430, P9010, P9011; diagnosis code V58.2x). As noted by Sridhar et al, “since there is no universal defining [diagnosis] code for pacemaker haematoma formation, we did our best to identify the [diagnosis] codes which are most consistent with haematoma formation.”^12^ Therefore, the codes used in the present study define bleeding in a broad manner, ie, all‐cause bleeding with no ability to stratify by event severity. This study varies from the prior analysis by Sridhar et al in that we did not include the diagnosis codes 996.60 to 996.65 (“mechanical complication of cardiac device implant and graft”) in our definition of hematoma, as we feel these are too broad to reliably identify a bleeding complication. Bleeding events during follow‐up were defined as any inpatient or outpatient visit within 30 days of the index visit with any diagnosis or procedure code listed above to define bleeding, excluding codes for transfusion procedures (because these could be related to various complications in follow‐up—not specifically related to the index CIED procedure). Thirty days was defined as the timeframe to identify bleeding‐related complications reasonably related to the index CIED procedure.

Infection complications were defined as any inpatient or outpatient visit for device infection in days 31 to 365 of follow‐up (diagnosis code 996.6 “infection and inflammatory reaction due to internal prosthetic device implant or graft; or procedure code 10180 “incision and drainage of postoperative wound infection”). Identification of infection was limited to this later follow‐up time period in order to evaluate whether early development (first 30 days) of bleeding complications was related to later development of device infection. Additionally, we evaluated whether the follow‐up visit for infection required complete device removal, using a codes list adapted from the Physician Quality Reporting System Measure 393—infection within 180 days of CIED Implantation, Replacement, or Revision (Table S3).

An estimate of the healthcare payer burden of bleeding‐related complications was summarized with the total visit payment made for an inpatient or outpatient visit during 30 days follow‐up for bleeding‐related complications. Total visit payment was defined as total payments to all providers by the primary payer, which is the sum of the insurance payment, coinsurance, copayments, and deductibles paid by the patient. The incremental index visit cost among patients with a bleeding complication was not summarized due to small sample sizes for patients with bleeding identified during the index visit. Additionally, only payment for the first follow‐up visit with a bleeding‐related complication was summarized; among patients with at least 1 visit, the median number of visits was 1 (90th percentile of patients had 2 or more visits). All payment information was adjusted to 2013 United States dollars (USD) using the medical care component of the consumer price index.

### Data Analysis

Statistical analyses were performed using the Instant Health Data Suite (Boston Health Economics, Inc, Waltham, MA) and SAS software (Version 9.2, SAS Institute, Cary, NC) packages. Descriptive statistics included mean, standard deviation, median, and interquartile ranges for continuous measures and proportions for binary measures. Statistical significance testing was conducted using the chi‐squared (χ^2^) test for categorical variables; the Fisher exact test was used for categorical variables with cell counts less than 10, and the Wilcoxon Mann‐Whitney tests for continuous variables. A logistic regression model was run to evaluate factors significantly associated with increased risk of a bleeding‐related complication during index or 30 days follow‐up. Additionally, two Cox proportional hazards models controlling for patient age, sex, region, Charlson score, and CIED type were run to evaluate factors correlated with a bleeding or infection complication in follow‐up. Covariates were selected based on measures both available in the data set and reasonably empirically correlated with the complications of interest.

## Results

### Patient Demographics and History of Pharmaceutical Therapy

A total of 42 606 patients met the study inclusion criteria. Patient and hospital characteristics, summarized by device type, are shown in Table 1. Mean patient age ranged from 61.5 years among replacement ICD procedures to 76.0 among replacement pacemaker procedures. The proportion of male patients ranged from 54.0% among replacement pacemaker procedures to 74.6% among replacement ICD procedures, with the majority of patients in all cohorts residing in the Southern region of the United States.

**Table 1:** Demographic Characteristics

	Pacemaker Primary	Pacemaker Replacement	ICD Primary	ICD Replacement
N	15 266	11 611	7045	8684
Age, y				
18 to 64	23.3%	20.0%	62.2%	35.0%
65 to 74	22.2%	15.4%	21.9%	24.2%
75 to 84	38.2%	33.9%	14.3%	29.8%
85+	16.3%	30.7%	1.6%	11.1%
Mean (SD)	73.26 (12.24)	75.99 (13.89)	61.54 (12.26)	69.65 (12.88)
Median	76	79	61	71
Male, %	60.9	54.0	74.5	74.6
Residence, %				
Northeast	12.6	19.4	15.6	19.7
South	40.1	32.7	43.4	34.6
Midwest	31.2	30.6	31.7	32.1
West	16.1	17.3	9.3	13.6
Charlson score group				
0	26.1%	26.1%	6.9%	10.5%
1	23.9%	21.3%	17.5%	19.7%
2	18.4%	18.0%	23.4%	19.7%
3+	31.7%	34.5%	52.2%	50.0%
Selected Charlson comorbidities				
Diabetes mellitus without complications	28.4%	29.7%	37.8%	38.7%
Diabetes mellitus with complications	7.7%	9.3%	10.3%	12.3%
Cardiovascular disease	25.5%	21.4%	19.4%	18.6%
Renal disease	13.0%	15.8%	14.5%	20.6%

ICD indicates implantable cardioverter‐defibrillator; SD, standard deviation.

The proportion of patients with any prescription fill for warfarin monotherapy within 90 days prior to the index CIED procedure ranged from 10.2% for primary ICDs to 17.8% for replacement pacemakers; the proportion with novel oral anticoagulant monotherapies ranged from 2.5% for primary ICDs to 4.8% for primary pacemakers (Table 2). The proportion with antiplatelet monotherapy (excluding aspirin) ranged from 7.3% for pacemaker replacements to 18.1% for primary ICDs. The proportion with concurrent warfarin and antiplatelet therapy ranged from 0.9% among replacement pacemakers to 2.7% among primary ICDs.

**Table 2:** History of Anticoagulant or Antiplatelet Prescription in 90 Days Prior to Pacemaker or ICD Procedure

	Pacemaker Primary	Pacemaker Replacement	ICD Primary	ICD Replacement
N	15 266	11 611	7045	8684
Therapy, %				
Warfarin monotherapy	14.7	17.8	10.2	17.5
Novel oral anticoagulant monotherapy^a^	4.8	3.4	2.5	2.9
Antiplatelet monotherapy^b^	8.6	7.3	18.1	10.7
Concurrent warfarin and antiplatelet therapy	1.3	0.9	2.7	1.4

ICD indicates implantable cardioverter‐defibrillator.

a Apixaban, rivaroxaban, or dabigatran.

b Abciximab, eptifibatide, tirofiban, cilostazol, clopidogrel, dipyridamole, prasugrel, ticagrelor, ticlopidine, or vorapaxar.

### Bleeding Complications

**Figure 2:**
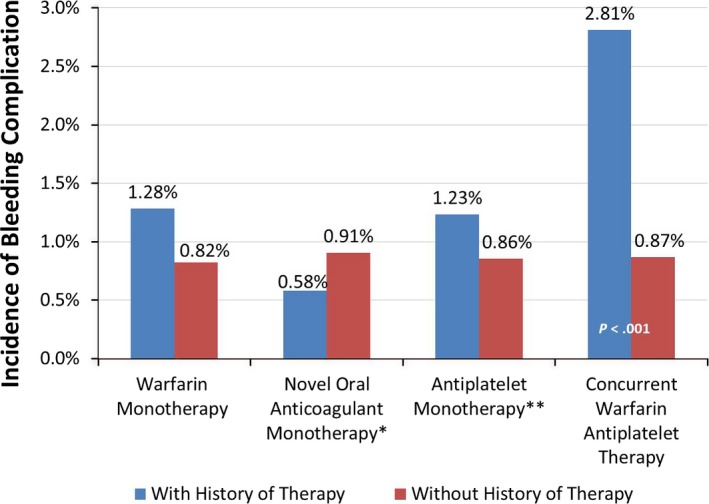
Incidence of bleeding during index visit or 30‐day follow‐up, by history of pharmaceutical therapy. *P*‐values: chi‐squared test for incidence of bleeding with therapy vs without (Fisher exact test where cell sample sizes <30). *Apixaban, rivaroxaban, or dabigatran. **Abciximab, eptifibatide, tirofiban, cilostazol, clopidogrel, dipyridamole, prasugrel, ticagrelor, ticlopidine, or vorapaxar.

The overall incidence of bleeding events recorded during the index visit through 30‐day follow‐up was 0.89%; by device type it was 0.71% for primary pacemakers, 0.85% for replacement pacemakers, 0.87% for primary ICDs, and 1.30% for replacement ICDs. The incidence of bleeding varied by history of pharmaceutical therapy, with patients with history of warfarin monotherapy, antiplatelet monotherapy, or concurrent warfarin and antiplatelet therapy having significantly greater incidence of bleeding complications than those with no history (all *P*<0.05; Figure 2). The difference in the incidence of bleeding complications was greatest among those with concurrent warfarin and antiplatelet therapy versus patients with no therapy (2.81% vs 0.87%, *P*<0.001). Patients with versus those without novel oral anticoagulant monotherapy had a similar incidence of bleeding (*P*=0.365); however, the sample size in this group was small (N=9 patients with history of oral anticoagulant therapy).

Overall, among patients with a bleeding‐related visit within 30 days of the initial CIED procedure, the majority of visits occurred in an outpatient (69%) versus inpatient setting (31%). Among patients with any follow‐up visit for a bleeding‐related complication, only 3.2% required complete device removal. The median (interquartile range) length of stay among patients with an inpatient visit for a bleeding‐related complication was 4.5 (3‐8) days. The three most frequent diagnoses listed during an inpatient visit for bleeding complications (other than hemorrhage or hematoma) were infection or inflammatory reaction due to internal prosthetic device, implant, or graft; atrial fibrillation and flutter; and systolic heart failure. Similarly, the three most frequent diagnoses during an outpatient visit for bleeding were hypertension, other forms of chronic ischemic heart disease, and complications due to internal prosthetic device implant or graft.

The median total visit payment for a bleeding‐related complication occurring in follow‐up in the outpatient setting ranged from $330 to $470 dependent on procedure type; those requiring an inpatient hospitalization ranged from $7341 to $17 445 (Table 3).

**Table 3:** Total Visit Cost (USD) for a Bleeding‐Related Complication Occurring Within 30 Days

	Follow‐Up: Outpatient Visit for Bleeding Complication	Follow‐Up: Inpatient Visit for Bleeding Complication
Primary pacemaker, N	63	29
Mean (SD)	$992 ($1378)	$14 473 ($12 397)
25th percentile	$141	$6938
Median	$330	$12 037
75th percentile	$1243	$19 775
Replacement pacemaker, N	57	25
Mean (SD)	$1024 ($1480)	$20 893 ($24 694)
25th percentile	$214	$7335
Median	$470	$11 587
75th percentile	$1296	$19 367
Primary ICD, N	41	15
Mean (SD)	$1079 ($2007)	$41 210 ($66 865)
25th percentile	$234	$13 569
Median	$369	$17 445
75th percentile	$944	$40 462
Replacement ICD, N	69	33
Mean (SD)	$1155 ($3130)	$15 627 ($20 419)
25th percentile	$163	$5843
Median	$350	$7341
75th percentile	$1174	$13 913

ICD indicates implantable cardioverter‐defibrillator; SD, standard deviation; USD, United States dollars.

### Infection

**Figure 3:**
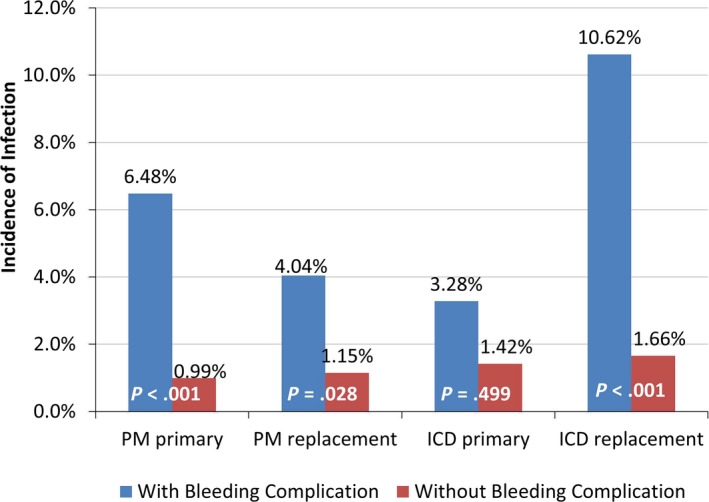
Incidence of infection during days 31 to 365 of follow‐up, overall and by presence of a bleeding complication in the first 30 days of follow‐up. *P*‐values: chi‐squared test for incidence of bleeding with therapy vs without (Fisher exact test where cell sample sizes <30). ICD indicates implantable cardioverter‐defibrillator; PM, pacemaker.

Overall, the incidence of infection from days 31 to 365 of follow‐up was 1.29%. Incidence of infection was significantly greater among patients with a bleeding‐related complication during index or the first 30 days of follow‐up compared to those without a bleeding complication for all device types except primary ICDs (pacemaker primary 6.5% vs 1.0%, *P*<0.001; pacemaker replacement 4.0% vs 1.2%, *P*=0.028; ICD primary 3.3% vs 1.4%, *P*=0.499; ICD replacement 10.6% vs 1.7%, *P*<0.001; Figure 3). Mean (standard deviation) time to infection was 92 (96) days following the index CIED procedure; median time to infection was 52.5 days.

Among patients presenting during days 31 to 365 of follow‐up with diagnosis of infection (including infection or inflammatory reaction due to cardiac device, implant, or graft; bacteremia, or other infection due to medical care), visits were almost evenly split between the inpatient (55%) and outpatient (45%) settings. Among those with an inpatient visit, the median (interquartile range) length of stay was 10 (5‐22) days. Overall, 19% of patients presenting with infection required complete device removal (note: visits for infection included severe infection or inflammatory reactions and mild postoperative wound infection requiring incision with drainage). Regarding inpatient visits for infection, the 3 most frequent diagnoses (other than infection) were combined systolic and diastolic heart failure, other forms of chronic ischemic heart disease, and congestive heart failure. Similarly, with outpatient visits for infection, the 3 most frequent diagnoses were atrioventricular block, other specified cardiac dysrhythmias, and congestive heart failure.

### Predictors of Bleeding or Infection Complications

Age 85 or older, a recent history of warfarin monotherapy, antiplatelet monotherapy, or concurrent warfarin and antiplatelet therapy, and Charlson scores of 2 or greater were all correlated with greater odds of a bleeding complication during the index CIED procedure visit or during 30 days follow‐up (all *P*<0.05; Table 4). Similar to univariate analyses, history of concurrent warfarin and antiplatelet therapy had the greatest effect on risk of a bleeding‐related complication (odds ratio [OR]=3.46; 95% CI 1.98‐5.64, *P*<0.001). In a separate Cox proportional hazards model controlling for age, sex, region, Charlson score, and CIED type, evidence of a bleeding complication during index or the first 30 days of follow‐up was correlated with increased risk of device infection in days 31 to 365 of follow‐up (hazard ratio [HR]=2.97; 95% CI 1.94‐4.54, *P*<0.001; Table 5).

**Table 4:** Logistic Regression: Predictors of Bleeding Complications During the Index Visit or 30‐Day Follow‐Up

Parameter	Odds Ratio	95% Confidence Interval	*P* Value
Demographics			
Age (relative to 18‐64), y			
65 to 74	0.99	0.71 to 1.37	0.937
75 to 84	1.31	0.98 to 1.76	0.068
85+	1.52^a^	1.08 to 2.13^a^	0.015^a^
Male	0.99	0.79 to 1.24	0.931
Region of US (relative to Midwest)			
Northeast	0.97	0.71 to 1.3	0.819
South	0.74^a^	0.57 to 0.96^a^	0.026^a^
West	1.18	0.87 to 1.58	0.290
History of pharmaceutical therapy			
Warfarin monotherapy	1.64^a^	1.26 to 2.12^a^	0.0002^a^
Novel oral anticoagulant monotherapy^b^	0.82	0.39 to 1.52	0.569
Antiplatelet monotherapy^c^	1.57^a^	1.15 to 2.12^a^	0.004^a^
Concurrent warfarin and antiplatelet therapy	3.46^a^	1.98 to 5.64^a^	<0.0001^a^
Charlson score (relative to 0)			
1	1.47	0.97 to 2.25	0.074
2	1.97^a^	1.32 to 3^a^	0.001^a^
≥3	2.22^a^	1.55 to 3.29^a^	<0.0001^a^
Generator type (relative to primary ICD)			
Primary pacemaker	0.86	0.61 to 1.23	0.399
Replacement pacemaker	0.93	0.65 to 1.35	0.708
Replacement ICD	1.39	1 to 1.95	0.054

ICD indicates implantable cardioverter‐defibrillator.

a Significant at the 95% confidence level.

b Apixaban, rivaroxaban, or dabigatran.

c Abciximab, eptifibatide, tirofiban, cilostazol, clopidogrel, dipyridamole, prasugrel, ticagrelor, ticlopidine, or vorapaxar.

**Table 5:** Cox Proportional Hazards Model: Predictors of Infection Occurring in Days 31 to 365 of Follow‐Up

	Hazard Ratio	95% Confidence Interval	*P* Value
Demographics			
Age (relative to 18‐64), y			
65 to 74	0.89	0.7 to 1.14	0.359
75 to 84	0.91	0.73 to 1.14	0.424
85+	0.97	0.72 to 1.31	0.832
Male	0.85	0.7 to 1.03	0.102
Region of United States (relative to Midwest)			
Northeast	1.08	0.83 to 1.42	0.556
South	1.08	0.87 to 1.33	0.475
West	1.10	0.84 to 1.44	0.491
Charlson score (relative to 0)			
1	0.94	0.68 to 1.3	0.712
2	0.76	0.55 to 1.05	0.097
≥3	0.85	0.64 to 1.13	0.257
Generator type (relative to primary ICD)			
Primary pacemaker	1.00	0.76 to 1.32	0.986
Replacement pacemaker	0.98	0.73 to 1.31	0.900
Replacement ICD	1.19	0.91 to 1.55	0.196
Bleeding complication during index visit or first 30 days follow‐up	2.97^a^	1.94 to 4.54^a^	<0.001^a^

ICD indicates implantable cardioverter‐defibrillator.

a Significant at the 95% confidence level.

## Discussion

Much of the current literature regarding bleeding in the perioperative period of CIED procedures has focused on variations in the relationship among hematoma, preoperative anticoagulant therapy regimens, and strategies for bridging heparin therapy. Few studies have focused on the relationship between hematoma and infection using nonrandomized data sets. Our present study served to provide a lower bound of the estimate of the real‐world incidence of hematoma following CIED procedures and to quantify the association between pocket hematoma and subsequent infection.

In the present study the incidence of bleeding from the initial visit through 30 days follow‐up ranged from 0.6% among patients with a history of novel oral anticoagulant therapy to 2.8% among those with concurrent warfarin and antiplatelet therapy. In comparison, results from BRUISE CONTROL, which compared strategies of heparin bridging versus continued warfarin treatment up to the time of a pacemaker or ICD procedure, showed that the incidence of clinically meaningful hematoma requiring incision and drainage was significantly greater in the heparin‐bridging group versus continuous warfarin (16.0% vs 3.5%, *P*<0.001).^9^ Additionally, the proportion of patients with hematoma requiring a prolonged hospital stay (defined as an increase of at least 1 day) was significantly greater with bridging than with the continuous warfarin strategy (4.7% vs 1.2%, *P*=0.006).^9^ One‐year follow‐up results of this study showed significantly greater incidence of device‐related infection among patients who developed hematoma versus those without (11.0% vs 1.5%, *P*<0.001).^14^ Further, findings from a meta‐analysis of 19 studies comparing continuous anticoagulation with heparin bridging found similar results to BRUISE CONTROL, with the incidence of bleeding events ranging from 2.2% among those with no anticoagulant therapy to 14.6% with heparin bridging.^8^ Although our results differ, the findings from our study may represent the best lower bound for the true incidence of pocket hematoma in real‐world practice. It is important to note that our results are not directly comparable to those of BRUISE CONTROL as only a minority of patients in our study had a history of an anticoagulant or antiplatelet medication fill in the 90 days prior to surgery (1.3% to 14.7%). However, the trend of greater incidence of bleeding among patients with history of warfarin therapy was upheld in our study, as was the finding of a significant association between the development of pocket hematoma and device infection, now with nonrandomized real‐world data.

In 1 of the only studies to date estimating the resource use associated with pocket hematoma, Sridhar et al performed a retrospective database analysis of the US Nationwide Inpatient Sample.^12^ These authors evaluated primary pacemaker and ICD procedures, and the incidence of hematoma ranged from 1.1% for single‐chamber pacemakers to 2.9% for biventricular ICDs. Patients who developed pocket hematoma had significantly longer inpatient length of stay than those without (8.7 days vs 4.8 days, *P*<0.001), which was associated with significantly higher total insurer payment ($48 815 vs $34 324, *P*<0.001). Incidence of pocket hematoma reported in that retrospective database was somewhat higher than that in the present study, as Sridhar et al defined bleeding events with a broader set of codes, specifically, the diagnosis codes 996.60 to 996.65 (“mechanical complication of cardiac device implant and graft”). Comparatively, we limited definition of hematoma to diagnosis codes 998.11 to 998.13 plus procedure codes for evacuation of the pocket or transfusion; we believe this to be more specific to a bleeding event. To the best of our knowledge, the analysis by Sridhar et al. is the only other claims database analysis of the incidence of bleeding following CIED procedures, with the remainder of the literature focused on retrospective case reports, prospective studies, or randomized controlled trials.

There are several limitations to this study, principally the underestimation of the true incidence of bleeding complications due to the lack of specificity in coding. Additionally, because we only identified patients undergoing CIED procedures in the outpatient setting, we by definition excluded high‐risk patients who may have had these procedures performed in an inpatient setting. This contributes to our estimates of the incidence of bleeding‐related complications as conservative lower bounds. Furthermore, because the data set was limited to medical claims data and contained no clinical details, it was not possible to segment bleeding events by severity. In addition, although we were able to identify patients with evidence of perioperative anticoagulant or antiplatelet therapy, this identification was based solely on prescription fills. No information was available regarding the timing of any preoperative discontinuations or the use of bridging therapy. Ideally, we would have had more detailed information on medication starts and stops in order to apply more specific risk stratification; however, this electronic health record level information was not available. Third, our estimates of the payments associated with a bleeding event were limited due to sample size constraints. We did not attempt to estimate the incremental payments for a bleeding event occurring during the index visit (similar to the analysis by Sridhar et al) because of the low incidence of bleeding observed during the index visit itself. We speculate that patients requiring only an outpatient visit for bleeding during follow‐up had lesser‐severity bleeding than patients with an inpatient visit for bleeding; however, there is no way to verify this without specific electronic health record information. Further research on the specific facility cost burden related to bleeding complications is warranted. Finally, we chose to limit the analysis to patients with only minor to moderate procedural complexity by selecting pacemaker and ICD procedures performed in an outpatient setting (as opposed to system upgrades, cardiac resynchronization therapy procedures, or procedures that had to be performed on an inpatient basis). We felt that in this more severely ill patient population it would be difficult to summarize resource utilization specifically related to the initial CIED procedure which would warrant a separate study.

In conclusion, this large, nationally representative, retrospective medical claims database analysis provides an improved real‐world estimate of the lower bound of incidence of bleeding during and in the 30 days following a CIED generator procedure as well as the association between a bleeding event and device infection.

## Sources of Funding

This study was funded by Medtronic Advanced Energy.

## Disclosures

Nichols and Vose disclose that they are employees of Medtronic Advanced Energy.

## Supporting information


**Table S1.** Codes for Patient Selection
**Table S2.** History of CIED Device Codes
**Table S3.** Codes Indicating Complete Device Removal*
