# Supplementary material for: Incidence of Bleeding‐Related Complications During Primary Implantation and Replacement of Cardiac Implantable Electronic Devices
Source: J Am Heart Assoc. 2017 Jan 22;6(1):e004263. doi: 10.1161/JAHA.116.004263 (PMC5523626; doi:10.1161/JAHA.116.004263)
Supplement: Supplementary file 1 — Table S1. Codes for Patient Selection Table S2. History of CIED Device Codes Table S3. Codes Indicating Complete Device Removal* [file JAH3-6-e004263-s001.pdf]

Supplemental Material

Table S1. Codes for Patient Selection

Cohort	Codes
Primary PM	CPT: 33206 or 33207 or 33208 (without 33225)<br>OR<br>ICD-9 proc: 37.81, 37.82, 37.83
Primary ICD	CPT: 33249 (without 33225)<br>OR<br>ICD-9 proc: 37.96
Replacement PM	CPT: 33227 or 33228 or 33229<br>OR<br>ICD-9 proc: 37.85, 37.86, 37.87
Replacement ICD	CPT: 33262 or 33263 or 33264<br>OR<br>ICD-9 proc: 37.98

Table S2. History of CIED Device Codes

Code Type	Code	Category	Description
**Require ≥ 1 device monitoring, history code in one year baseline to define a "replacement" procedure. If no code for device monitoring present during baseline, then a "primary" procedure**			
CPT	93279	Device monitoring	Programming device evaluation (in person) with iterative adjustment of the implantable device to test the function of the device and select optimal permanent programmed values with analysis, review and report by a physician or other qualified health care professional; single lead pacemaker system
CPT	93280	Device monitoring	Programming device evaluation (in person) with iterative adjustment of the implantable device to test the function of the device and select optimal permanent programmed values with analysis, review and report by a physician or other qualified health care professional; dual lead pacemaker system
CPT	93281	Device monitoring	Programming device evaluation (in person) with iterative adjustment of the implantable device to test the function of the device and select optimal permanent programmed values with analysis, review and report by a physician or other qualified health care professional; multiple lead pacemaker system
CPT	93282	Device monitoring	Programming device evaluation (in person) with iterative adjustment of the implantable device to test the function of the device and select optimal permanent programmed values with analysis, review and report by a physician or other qualified health care professional; single lead transvenous implantable defibrillator system
CPT	93283	Device monitoring	Programming device evaluation (in person) with iterative adjustment of the implantable device to test the function of the device and select optimal permanent programmed values with analysis, review and report by a physician or other qualified health care professional; dual lead transvenous implantable defibrillator system
CPT	93284	Device monitoring	Programming device evaluation (in person) with iterative adjustment of the implantable device to test the function of the device and select optimal permanent programmed values with analysis, review and report by a physician or other qualified health care professional; multiple lead transvenous implantable defibrillator system
CPT	93286	Device monitoring	Peri-procedural device evaluation (in person) and programming of device system parameters before or after a surgery, procedure, or test with analysis, review and report by a physician or other qualified health care professional; single, dual, or multiple lead pacemaker system
CPT	93287	Device monitoring	Peri-procedural device evaluation (in person) and programming of device system parameters before or after a surgery, procedure, or test with analysis, review and report by a physician or other qualified health care professional; single, dual, or multiple lead implantable defibrillator system
CPT	93288	Device monitoring	Interrogation device evaluation (in person) with analysis, review and report by a physician or other qualified health care professional, includes connection, recording and disconnection per patient encounter; single, dual, or multiple lead pacemaker system
CPT	93289	Device monitoring	Interrogation device evaluation (in person) with analysis, review and report by a physician or other qualified health care professional, includes connection, recording and disconnection per patient encounter; single, dual, or multiple lead transvenous implantable defibrillator system, including analysis of heart rhythm derived data elements
CPT	93294	Device monitoring	Interrogation device evaluation(s) (remote), up to 90 days; single, dual, or multiple lead pacemaker system with interim analysis, review(s) and report(s) by a physician or other qualified health care professional
CPT	93295	Device monitoring	Interrogation device evaluation(s) (remote), up to 90 days; single, dual, or multiple lead implantable defibrillator system with interim analysis, review(s) and report(s) by a physician or other qualified health care professional
CPT	93296	Device monitoring	Interrogation device evaluation(s) (remote), up to 90 days; single, dual, or multiple lead pacemaker system or implantable defibrillator system, remote data acquisition(s), receipt of transmissions and technician review, technical support and distribution of results

Code Type	Code	Category	Description
CPT	93642	Device monitoring	Electrophysiologic evaluation of single or dual chamber transvenous pacing cardioverter-defibrillator (includes defibrillation threshold evaluation, induction of arrhythmia, evaluation of sensing and pacing for arrhythmia termination, and programming or reprogramming of sensing or therapeutic parameters)
CPT	93724	Device monitoring	Electronic analysis of antitachycardia pacemaker system (includes electrocardiographic recording, programming of device, induction and termination of tachycardia via implanted pacemaker, and interpretation of recordings)
CPT	93299	Device monitoring	Interrogation device evaluation(s), (remote) up to 30 days; implantable cardiovascular monitor system or implantable loop recorder system, remote data acquisition(s), receipt of transmissions and technician review, technical support and distribution of results
CPT	93731	Device monitoring	93280 after 12/31/2009
CPT	93732	Device monitoring	93280 after 12/31/2009
CPT	93733	Device monitoring	93293 after 2009
CPT	93293	Device monitoring	Transtelephonic rhythm strip pacemaker evaluation(s) single, dual, or multiple lead pacemaker system, includes recording with and without magnet application with analysis, review and report(s) by a physician or other qualified health care professional, up to 90 days
CPT	93734	Device monitoring	93288 after 2009
CPT	93735	Device monitoring	93279 after 2009
CPT	93736	Device monitoring	93293 after 2009
CPT	93741	Device monitoring	93289 after 2009
CPT	93742	Device monitoring	93282 after 2009
CPT	93743	Device monitoring	93289 after 2009
CPT	93744	Device monitoring	93283 or 93284 after 2009
ICD9 Dx	V45.01	History of device	Cardiac pacemaker in situ
ICD9 Dx	V45.02	History of device	Automatic implantable cardiac defibrillator in situ
ICD9 Dx	V53.31	History of device	Fitting and adjustment of cardiac pacemaker
ICD9 Dx	V53.32	History of device	Fitting and adjustment of automatic implantable cardiac defibrillator
ICD9 Proc	89.45	Device monitoring	Artificial pacemaker rate check
ICD9 Proc	89.46	Device monitoring	Artificial pacemaker artifact wave form check
ICD9 Proc	89.47	Device monitoring	Artificial pacemaker electrode impedance check
ICD9 Proc	89.48	Device monitoring	Artificial pacemaker voltage or amperage threshold check

Code Type	Code	Category	Description
ICD9 Proc	89.49	Device monitoring	Automatic implantable cardioverter/defibrillator (AICD) check
ICD9 Proc	37.20	Device monitoring	Noninvasive programmed electrical stimulation [NIPS]

Table S3. Codes Indicating Complete Device Removal*

Code Type	Code	Description
ICD-9	00.50	Implantation of cardiac resynchronization pacemaker without mention of defibrillation, total system (CRT-P)
ICD-9	00.51	Implantation of cardiac resynchronization defibrillator, total system (CRT-D) 00.51
ICD-9	00.52	Implantation or replacement of transvenous lead (electrode) into left ventricular coronary venous system
ICD-9	00.53	Implantation or replacement of cardiac resynchronization pacemaker, pulse generator only (CRT-P) 00.53
ICD-9	00.54	Implantation or replacement of cardiac resynchronization defibrillator, pulse generator only (CRT-D) 00.54
ICD-9	37.80	Insertion of permanent pacemaker, initial or revision, type of device not specified
ICD-9	37.81	Initial insertion of single-chamber pacemaker device, not specified as rate responsive
ICD-9	37.82	Initial insertion of single-chamber pacemaker device, rate responsive
ICD-9	37.83	Initial insertion of dual-chamber pacemaker device
ICD-9	37.85	Replacement of any type pacemaker device with single chamber device, not specified as rate responsive
ICD-9	37.86	Replacement of any type pacemaker device with single chamber device, rate responsive
ICD-9	37.87	Replacement of any type pacemaker device with dual chamber device
ICD-9	37.89	Revision or removal of pacemaker device
ICD-9	37.94	Implantation or replacement of automatic cardioverter-defibrillator (AICD), total system
ICD-9	37.96	Implantation or replacement of automatic cardioverter-defibrillator pulse generator only
ICD-9	37.98	Replacement of automatic cardioverter-defibrillator (AICD), pulse generator only
CPT	33206	Insertion of new or replacement of permanent pacemaker with transvenous electrode(s); atrial
CPT	33207	Insertion of new or replacement of permanent pacemaker with transvenous electrode(s); ventricular
CPT	33208	Insertion of new or replacement of permanent pacemaker with transvenous electrode(s); atrial and ventricular
CPT	33212	Insertion or replacement of pacemaker pulse generator only; single-chamber, atrial or ventricular 33212
CPT	33213	Insertion or replacement of pacemaker pulse generator only; dual-chamber 33213
CPT	33214	Upgrade of implanted pacemaker system, conversion of single chamber system to dual chamber system (includes removal of previously placed pulse generator, testing of existing lead, insertion of new lead, insertion of new pulse generator)
CPT	33216	Insertion of a single transverse electrode, permanent pacemaker or cardioverter-defibrillator
CPT	33217	Insertion of 2 tranvenous electrodes, permanent pacemaker or cardioverter-defibrillator 33217
CPT	33224	Insertion of pacing electrode, cardiac venous system, for left ventricular pacing, with attachment to previously placed pacemaker or pacing cardioverter-defibrillator pulse generator (including revision of a pocket, removal, insertion, and/or replacement of generator
CPT	33225	Insertion of pacing electrode, cardiac venous system, for left ventricular pacing, at time of insertion of pacing cardioverter-defibrillator or pacemaker pulse generator (including upgrade to dual-chamber system) (Use in conjunction with code for primary procedure)
CPT	33226	Repositioning of previously implanted cardiac venous system (left ventricular) electrode (including removal, insertion, and/or replacement of generator)

Code Type	Code	Description
CPT	33227	Removal and replacement of permanent pacemaker pulse generator; single lead system
CPT	33228	Removal and replacement of permanent pacemaker pulse generator; dual lead system 33228
CPT	33229	Removal and replacement of permanent pacemaker pulse generator; multiple lead system 33229
CPT	33233	Removal of permanent pacemaker pulse generator 33233
CPT	33240	Insertion of single or dual chamber pacing cardioverter-defibrillator pulse generator 33240
CPT	33249	Insertion or replacement of permanent pacing cardioverter-defibrillator system with transvenous lead(s), single or dual chamber
CPT	33262	Removal and replacement of pacing cardioverter-defibrillator pulse generator; single lead system
CPT	33263	Removal and replacement of pacing cardioverter-defibrillator pulse generator; dual lead system 33263
CPT	33264	Removal and replacement of pacing cardioverter-defibrillator pulse generator; multiple lead system 33264
HCPCS	G9410	Patient admitted within 180 days, status post cied implantation, replacement, or revision with an infection requiring device removal or surgical revision
HCPCS	G9412	Patient Admitted Within 180 Days, Status Post Cied Implantation, Replacement, Or Revision With An Infection Requiring Device Removal Or Surgical Revision

*This codes list was adapted from the PQRS measure 393: Infection within 180 days of CIED Implantation, Replacement, or Revision
